# Vitamin D Deficiency Is Common in Ghana despite Abundance of Sunlight: A Multicentre Comparative Cross-Sectional Study

**DOI:** 10.1155/2021/9987141

**Published:** 2021-06-10

**Authors:** Samuel Asamoah Sakyi, Maxwell Hubert Antwi, Linda Ahenkorah Fondjo, Edwin Ferguson Laing, Richard K. Dadzie Ephraim, Alexander Kwarteng, Benjamin Amoani, Seth Christopher Appiah, Bright Oppong Afranie, Stephen Opoku, Tonnies Abeku Buckman

**Affiliations:** ^1^Department of Molecular Medicine, School of Medicine and Dentistry, Kwame Nkrumah University of Science and Technology, Kumasi, Ghana; ^2^Department of Medical Laboratory Sciences, Faculty of Allied Health, University of Cape Coast, Cape Coast, Ghana; ^3^Department of Biochemistry and Biotechnology, Kwame Nkrumah University of Science and Technology, Kumasi, Ghana; ^4^Department of Biomedical Science, School of Allied Health Sciences, University of Cape Coast, Cape Coast, Ghana; ^5^Center for International Health, University of Munich Medical, Ludwig-Maimillians Universitate of Munchen, Munchen, Germany; ^6^Department of Medical Diagnostics, Faculty of Allied Health Sciences, College of Health Sciences, Kwame Nkrumah University of Science and Technology, Kumasi, Ghana

## Abstract

**Background:**

Vitamin D is a steroid hormone important for the normal functioning of the body. It is produced through skin exposure to sunlight and from the diet. Although Ghana is located in the tropics where sunlight is abundant, factors like culture, diet, skin pigmentation, variation in the ozone layer, and geographical area influence the optimization of vitamin D concentration. It is imperative to evaluate the interplay between sunshine exposure, proinflammatory cytokines, and mediators of vitamin D metabolism and their relationship to vitamin D status in three geographical sections among apparent healthy Ghanaians.

**Methods and Results:**

In a cross-sectional study, a total of five hundred (500) healthy blood donors from three geographical areas in Ghana were enrolled. Their age ranged from 17 to 55 years with a mean age of 27.97 ± 8.87 years. The overall prevalence rate of vitamin D deficiency was 43.6% (218/500), with 41.2% (91/221), 45.3% (63/139), and 45.7% (64/140) of vitamin D deficiency being recorded in participants from the Northern Sector (NS), Middle Belt (MB), and Southern Sector (SS), respectively. However, there were no significant differences in the proportions of vitamin D deficiency across various geographical sectors. The median 25-hydroxyvitamin D serum levels were compared among geographical areas (NS, MB, and SS) and there were no significant differences (*P*=0.275) after adjusting for confounding factors. 25-Hydroxyvitamin D correlated positively with corrected ionized calcium (rs = 0.622, *P* ≤ 0.001) and phosphorus (rs = 0.299, *P* ≤ 0.001) and negatively correlated with SBP (rs = −0.092, *P*=0.039), vitamin D binding protein (VDBP) (rs = −0.421, *P* ≤ 0.001), intact parathyroid hormone (iPTH) (rs = −0.0568, rs ≤ 0.001), IFN-gamma (rs = −0.684, *P* ≤ 0.001), and TNF-alpha (rs = −0.600, *P* ≤ 0.001). After adjusting for possible confounders, not having knowledge about vitamin D foods, taking fewer vitamin D foods, and higher levels of IF-*γ* and IL-10 were associated with a higher risk of having vitamin D deficiency.

**Conclusion:**

The prevalence of 25-hydroxyvitamin D deficiency is high among the general adult population in Ghana despite the abundance of sunlight. Increasing knowledge on vitamin D diet coupled with a daily intake of vitamin D dietary supplements is likely to reduce the risk of developing 25-hydroxyvitamin D deficiency.

## 1. Introduction

The “sunshine” vitamin, also called vitamin D, is a steroid hormone that is essential for the normal functioning of the body including the intestine, skin, bone, parathyroid glands, immune system, pancreas, and the healthy growth of a developing fetus [1]. 25-Hydroxyvitamin D deficiency has been identified as a global challenge in both healthy and unhealthy populations, and it is estimated that up to one billion healthy individuals are living with hypovitaminosis D globally [2]. Vitamin D produced from sunlight through skin exposure and from the diet is converted into 25-hydroxyvitamin D [25(OH)D] in the liver and is carried into the circulation by vitamin D binding protein to the kidney [1, 3, 4]. Vitamin D status is determined more by exposure of the skin to sunlight than by dietary intake of the vitamin, because food sources are relatively limited, particularly when the food supply is not fortified with vitamin D [5]. Vitamin D is one of the many nutrients our bodies need to stay healthy and is introduced to the body in two forms which are vitamin D_3_ and D_2_ [6]. Vitamin D_3_ is produced in the skin by ultraviolet B radiation (UVB) from sunlight and vitamin D_2_ is obtained in the diet from selected food sources, such as deep-sea fatty fish or egg yolk [7].

Vitamin D deficiency varies highly across regions, with the prevalence ranging between 30 and 90% [8]. A steady measure of getting vitamin D is endogenous skin synthesis through UVB light exposure at wavelengths of 290–315 nm [9] and it is estimated that sun exposure on bare skin for 5–10 minutes twice per week allows the body's ability to produce sufficient vitamin D [10]. The kidney converts (25OHD) into the active form called 1, 25-dihydroxyvitamin D (1, 25(OH) 2D) through the activity of the enzyme 1-alpha hydroxylase (CYP27B1) whose function depends on parathyroid hormone (PTH), calcium, and phosphorous levels (33-34). It is thus important to assess the interplay between PTH, kidney function, calcium, and phosphorus levels amid adequate sunshine vis-a-vis vitamin D status. Tissues that are receptive to vitamin D express the vitamin D receptor gene (VDR) and 1-alpha hydroxylase (CYP27B1) enzymes and include many cells of the immune system (35).

The activity of the 1-alpha hydroxylase (CYP27B1) enzyme to produce local 1, 25(OH) 2D (calcitriol) in the immune environment depends on proinflammatory cytokines like TNF-alpha, IL6, and IFY (36–39).

Although Ghana is located in the tropics where sunlight is abundant, factors like culture (especially our style of dressing), diet, skin pigmentation, variation in the ozone layer, and geographical area (latitude) influence the optimization of vitamin D concentration [11, 12]. In addition, data on vitamin D status in sub-Saharan Africa is limited, especially in Ghana. It is thus imperative to evaluate the interplay between sunshine exposure, proinflammatory cytokines, and mediators of vitamin D metabolism and their relationship to vitamin D status in three geographical sections among apparent healthy Ghanaians.

## 2. Materials and Methods

### 2.1. Study Design/Settings

This cross-sectional study was conducted in selected hospitals across seven regions of Ghana for 5 months. The selected hospitals were (a) Bolgatanga Regional Hospital, Nandowli Hospital, and Kpandai Hospital, which present the Northern Sector (NS); (b) Wenchi Methodist Hospital and St. Patrick's Hospital Offinso-Maase which present the Middle Belt (MB); (c), Effia Nkwanta Regional Hospital and Koforidua Regional Hospital which present the Southern Sector (SS) (Figure S1; Table S1). Ghana is geographically placed near the equator and lies between latitude: 7°57′9.97″N and longitude: −1°01′50.56″W. It occupies an area of about 240,000 km^2^ of the African continent and is the thirteenth most populated country in Africa and among the top five populated countries in West Africa, with more than 26 million people accounting for about 2.5% of African's population (Ghana Statistical Service, 2010 Population and Housing Census) [13].

### 2.2. Questionnaire and Data Collection

Structured validated questionnaires were administered to study participants to obtain their sociodemographic characteristics, knowledge on vitamin D-rich foods, and frequent intake of various vitamin D-rich foods. Information on their health status about the presence or absence of chronic disease conditions was also included in the questionnaire.

### 2.3. Study Population/Subjects Selection

Using a systematic sampling technique, a total of five hundred (500) healthy blood donors between the ages of 17 years and 60 years were selected. A systematic random sampling technique was used for sample collection as described (Table S2). Healthy blood donors who have passed screening and are free from any diseases including pulmonary tuberculosis, diabetes mellitus, malaria, sexually transmitted infections (STIs), and blood-borne infections were included in this study. Pregnant women, children, subjects with clinical records or symptoms showing chronic ailments such as renal, hypertension, and any other diseases, and subjects with drugs that could influence vitamin D intake and metabolism were all excluded from the study.

### 2.4. Sample Collection Processing and Measurement

Ten (10) milliliters (ml) of venous blood was taken from a prominent vein of each donor. Three (3) ml of blood was dispensed into dipotassium ethylenediaminetetraacetic acid (K2EDTA) tubes, one (1) ml into fluoride oxalate tubes, and five (5) ml into gel separator tubes (5 ml MICRO POINT clot activator tube; batch number: KJ040AS). K2EDTA blood samples were screened for hemoglobin levels, typhoid test, hepatitis B and hepatitis C, HIV/AIDs, syphilis, and malaria. A calibrated copper solution of standard hemoglobin concentrations of 13.0 g/dl and 12.5 g/dl for males and females, respectively, was the method used to determine the hemoglobin level of the blood donors. The tubes were positioned in the centrifuge holes (HERMLE Z300K, Labsource, Inc., Romeoville, IL 60446) and spun at 3500 rpm for about 8 minutes to get plasma and serum where appropriate. Blood collected into fluoride oxalate tubes was screened for diabetes. The serum was aliquoted into cryotubes and stored at −70°C (Thermo Scientific™ Revco™ UxF—Ultra-Low Temperature Freezers, USA) until the measurement of the biochemical assays.

### 2.5. Biochemical Assays

The serum concentrations of 25(OH)D, intact parathyroid hormone (iPTH), and vitamin D binding protein (VDBP), interleukin-10 (IL-10), tumor necrosis factor-alpha (TNF-alpha), and interferon-gamma (IFN-gamma) were measured with Inqaba Biotec ELISA plate reader (Inqaba Biotechnical Industries (Pty) Ltd., South Africa) using reagents from Biobase Biotech (Jinan) Co., Ltd., China, whose basic principle is Sandwich enzyme-linked immunosorbent assay (ELISA). In addition, serum concentrations of ionized calcium, phosphorus, albumin, and creatinine were measured with the Biotechnica BT 3500 Chemistry analyzer using reagents from BT Biotechnica Co., Ltd., Rome, Italy.

### 2.6. Classification of 25(OH)D Status

Serum 25(OH)D levels less than 20 ng/ml were considered vitamin D deficient and those greater than 20 ng/ml as not deficient [14].

### 2.7. Calculation of Corrected Ionized Calcium

Corrected ionized calcium was then calculated using the following formula:

(1) $$\begin{array}{c} \text{Corrected ionized Ca}\left(\text{mmol}/\text{l}\right)=\left[\text{calcium measured}\left(\text{mmol}/\text{l}\right)+0.02\left(40-\text{albumin measured}\left(\text{g}/\text{dl}\right)\right)\right]. \end{array}$$

### 2.8. Statistical Analysis

IBM SPSS version 20.0 statistics was used to generate and analyze data and clean for outrageous values at regular intervals. Categorical variables were presented and reported as frequencies with their corresponding percentages. Means with their standard deviation and medians (ranges) were used to summarize continuous variables. Comparison between two means of parametric and nonparametric continuous variables was done using independent sample *t*-test and Mann–Whitney *U* test, respectively. Chi-square test statistic was employed to determine any association between the categorical variables. Spearman's Rho statistic was used to determine the relationship between 25OHD and the biochemical parameters, while the odds of biochemical variables in predicting vitamin D deficiency were done using multivariate logistic regression analysis. A *P* value of less than 0.05 was considered statistically significant. Coefficients were then determined with their respective 95% confidence intervals.

## 3. Results

A total of five hundred (500) healthy blood donors were enrolled in this study, of which 72.4% were males and 27.6% were females. Their ages ranged from 17 to 55 years with a mean age of 27.97 ± 8.87 years. The age group with the highest proportion was 20–29 years, contributing 47.8% of all participants. A total of 41.4% of the participants had secondary school education and the majority of the participants were still single (61.8%). Almost 75.2% of the study participants are Christians and 43.4% were informally employed (Table 1).

**Figure 1:**
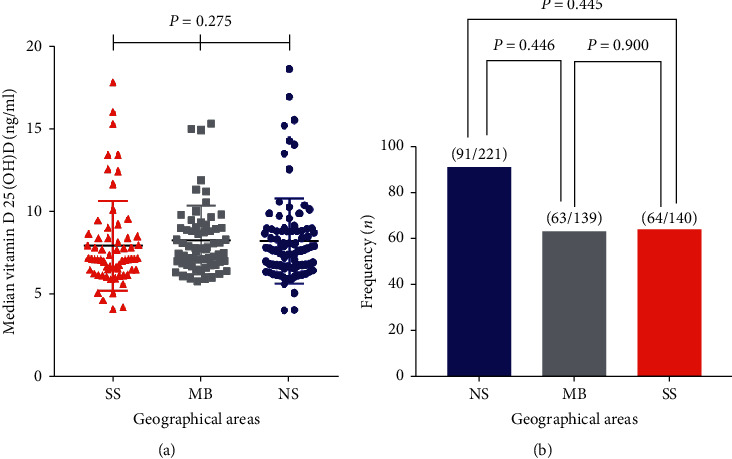
(a) Kruskal–Wallis test was used to compare the median of 25-hydroxyvitamin D serum levels among participants. (b) Prevalence of vitamin D deficiency among study participants. *P* value <0.05 is statistically significant, NS = Northern Sector, MB = Middle Belt, and SS = Southern Sector. *P* value for (a) is for across all the groups, other factors such as hemodynamics, biochemistry parameters, age, and weight were adjusted.

The median 25-hydroxyvitamin D serum levels were compared across geographical areas (NS, MB, and SS) and there were no significant differences (*P*=0.275) as confounding factors were adjusted (Figure 1(a)). The total prevalence rate of vitamin D deficiency was 43.6% (218/500) with 41.2% (91/221), 45.3% (63/139), and 45.7% (64/140) of vitamin D deficiency recorded for NS, MB, and SS, respectively (Figure 1(b)). However, there was no significant difference among the proportions of the prevalence of vitamin D deficiency among participants (NS vs. SS, *P*=0.455; NS vs. MB, *P*=0.446; SS vs. MB, *P*=0.900).

Serum 25-hydroxyvitamin D was correlated positively with corrected ionized calcium (*r* = 0.622, *P* ≤ 0.001) and phosphorus (*r* = 0.299, *P* ≤ 0.001) and negatively correlated with SBP (*r* = −0.092, *P*=0.039), VDBP (*r* = −0.421, *P* ≤ 0.001), IPTH (*r* = −0.568, *P* ≤ 0.001), IFN-gamma (*r* = −0.684, *P* ≤ 0.001), IL-10 (*r* = 0.575, *P* ≤ 0.001), and TNF-alpha (*r* = −0.600, *P* ≤ 0.001). Results are shown in Table 2.

Table 3 depicts the binary logistic regression analysis which predicted the odds ratio for risk factors of vitamin D deficiency among study participants. For both the crude and adjusted odds ratio, participants who did not know vitamin D foods were at a higher risk of having vitamin D deficiency (cOR = 2.93, *P*=0.014; aOR = 4.39, *P*=0.002). With the adjusted odds ratio, those who take in milk weekly have a higher chance of having vitamin D deficiency (aOR = 2.63, *P*=0.040). In addition, eating salmon monthly as compared to the rest makes the participants at higher risk of vitamin D deficiency (aOR = 1.96, *P*=0.032). Furthermore, those who only monthly take in fruits and vegetables are highly vulnerable to having vitamin deficiency status (aOR = 7.02, *P* < 0.001); see Tables 3 and S3.

## 4. Discussion

Ghana is a tropical country with a lot of sunlight; however, it is not a homogenous entity for geography, climate, water sources, food production and availability, or the religious and cultural practices, skin pigmentation, and burden of infectious and chronic diseases of its people. All these factors are likely to affect and/or be affected by vitamin D status [15]. We, therefore, investigated whether the abundance and variability of sunlight in Ghana necessarily confer adequate vitamin D optimization among apparent healthy Ghanaians in three geographical sections. We also explored the factors associated with vitamin D deficiency.

The results of the study confirm a high prevalence of vitamin D deficiency of 43.6% among the adult healthy population in Ghana. In a cross-sectional study among the healthy adult population of Isfahan in Iran, vitamin D deficiency (25OHD <20 ng/mL) was observed to be 50.8% which is higher than this current study. Although Isfahan is a sunny city, direct exposure to the sun is, however, limited because in most Muslim countries, all women are required to wear a scarf (Hajib) and long-sleeve clothes and they constituted 78.1% of their study participants. This why Iran has more severe vitamin D deficiency than Ghana which is mostly a Christian country [16, 17], even though Iran and Ghana are located in the subtropics (dry hot) and tropics (humid hot), respectively [18].

In this study, 41.2% (91/221), 45.3% (63/139), and 45.7% (64/140) of vitamin D deficiency rates were recorded for the Northern Sector (NS), Middle Belt (MB), and Southern Sector (SS) of Ghana, respectively, with no significant proportional differences even though there are variations in the latitudes and intensity of sunshine in these areas [13]. A cross-sectional study among the healthy seminomadic Fulani population in northern part of Nigeria by Glew et al. reported a vitamin D deficiency of 21.6%, which is lower than the rate observed in this current study for NS since both geographical areas have similar “high sunshine.” The observed difference could partly be explained by the fact that most of their study populations were cattle farmers tending to their cattle, thereby benefitting from extensive exposure of their skin to sunlight [19]. However, the majority of the NS participants in the current study were students or formally employed which kept them indoors. Consequently, it is not solely the abundance of sunshine that influences the optimization of vitamin D concentrations but rather the exposure of the skin to the ultraviolet ray of the sunlight [20]. Results from this study are inconsistent with a study by Gebreegziabher and Stoecker as they reported that about 84.2% of people situated in the southern part of Ethiopia were vitamin D deficient [21].

It was significantly observed in this study that most of the participants from the southern sector take dairy products frequently compared to participants from other geographical areas. Apart from this, there were equal proportions of frequencies recorded for the predisposing risk factors stratified among the three geographical areas [additional file, Table S2]. Results from this study show that intake of various vitamin D-rich foods independently gives one less chance of being vitamin D deficient. Moreover, consistently with previous studies, not knowing vitamin D foods [22], taking fewer vitamin D foods [23, 24], and higher levels of IF-*γ* and IL-10 [25] were associated with higher chances of having vitamin D deficiency.

From the correlation analysis, the results were similar to other studies as 25-hydroxyvitamin D was correlated positively with corrected ionized calcium and phosphorus [26] and negatively correlated to systolic blood pressure [27, 28], vitamin D binding protein, parathyroid hormone (PTH) [29, 30], interferon-gamma, and tumor necrosis factor-alpha [31].

In this study, therefore, the vitamin D deficiency determinants were controlled when comparing the serum levels of 25-hydroxyvitamin D across the geographical areas. In this comparative cross-sectional study, substantial variability in serum 25(OH)D concentrations does not exist despite the varying degree of abundant sun exposure, as median serum 25(OH)D levels of participants were significantly equal across geographical sections of Ghana (NS, MB, and SS). The limitation of the current study is our inability to measure the sun exposure habits of the respondents. However, this has no substantive effect on our main findings.

## 5. Conclusion

The prevalence of 25(OH)D deficiency is high among the general adult population in all three sectors of Ghana despite the abundance and variability in sunlight. Increased level skin exposure to sunlight coupled with a daily intake of vitamin D dietary supplements will reduce the risk of developing 25(OH)D deficiency.

## Figures and Tables

**Table 1:** Sociodemographic and baseline characteristics of study participants.

Variables	Total (*n* = 500)	Divisions			*P* value
		NS (*n* = 221)	MB (*n* = 139)	SS (*n* = 140)	
Age (years) (mean ± SD)	27.97 ± 8.87	27.19 ± 8.05	26.31 ± 7.02	30.83 ± 9.38	<0.001

Age group (years)					
<20	84 (16.8%)	44 (52.4%)	23 (27.4%)	17 (20.2%)	0.157
20–29	239 (47.8%)	101 (42.3%)	85 (35.5%)	53 (22.2%)	<0.001
30–39	121 (24.2%)	58 (47.9%)	17 (14.0%)	46 (38.1%)	<0.001
≥40	56 (11.2%)	18 (32.1%)	14 (25.0%)	24 (42.9%)	0.027

Gender					
Male	362 (72.4%)	154 (42.5%)	107 (29.6%)	101 (27.9%)	0.320
Female	138 (27.6%)	67 (61.0%)	32 (38.4%)	39 (38.6%)	0.320

Marital status					
Single	309 (61.8%)	138 (44.7%)	101 (32.7%)	70 (22.6%)	<0.001
Married	180 (36.0%)	80 (44.4%)	36 (20.0%)	64 (35.6%)	0.003
Divorced	11 (2.2%)	3 (27.3%)	2 (18.2%)	6 (54.5%)	0.14

Religious status					
Christians	376 (75.2%)	167 (44.4%)	105 (27.9%)	104 (27.7%)	0.957
Muslims	97 (19.4%)	41 (42.3%)	26 (26.8%)	30 (30.9%)	0.774
Traditionalists	27 (5.4%)	13 (48.1%)	8 (29.6%)	6 (22.3%)	0.475

Educational background					
None	69 (13.8%)	29 (42.0%)	30 (43.5%)	10 (14.5%)	0.002
Basic	76 (15.2%)	37 (48.7%)	12 (15.8%)	27 (35.3%)	0.032
Secondary	207 (41.4%)	95 (45.9%)	38 (18.4%)	74 (35.7%)	<0.001
Tertiary	148 (29.6)	60 (40.5%)	59 (39.9%)	29 (19.6%)	<0.001

Occupational status					
Unemployed	6 (1.2%)	2 (33.3%)		4 (66.7%)	0.078
Informal	217 (43.4%)	93 (42.9%)	58 (26.7%)	66 (30.4%)	0.573
Formal	64 (12.8%)	28 (43.8%)	9 (14.1%)	27 (42.2%)	0.001
Student	213 (42.6%)	98 (46.0%)	72 (33.8%)	43 (20.2%)	0.342
SBP (mmHg)	114.64 ± 6.52	114.34 ± 5.97	115.54 ± 7.34	114.21 ± 6.47	0.158
DBP (mmHg)	75.38 ± 5.38	75.57 ± 5.16	75.61 ± 5.66	74.86 ± 5.43	0.398
IPTH (pg/ml)	7.12 (6.24–8.67)	7.02 (5.95–8.77)	7.30 (6.33–8.67)	7.35 (6.38–8.62)	0.409
VDBP (ng/ml)	26.38 (8.46–53.23)	27.90 (8.38–61.67)	21.57 (7.97–46.22)	28.37 (9.90–28.77)	0.035
Corrected calcium (mmol/l)	2.20 ± 0.19	2.20 ± 0.19	2.17 ± 0.17	2.21 ± 0.20	0.166
Phosphorus (mmol/l)	1.30 ± 0.17	1.31 ± 0.18	1.28 ± 0.13	1.32 ± 0.18	0.143
Albumin (g/dl)	43.09 ± 2.90	43.27 ± 3.22	42.86 ± 2.62	43.04 ± 2.81	0.410
Creatinine (mmol/l)	99.52 ± 28.08	101.34 ± 29.18	94.92 ± 21.60	101.22 ± 31.45	0.075

*P* value <0.05 = statistically significant, SD = standard deviation, NS = Northern Sector, MB = Middle Belt, SS = Southern Sector, SBP = systolic blood pressure, DBP = diastolic blood pressure, IPTH = intact parathyroid hormone, and VDBP = vitamin D binding protein.

**Table 2:** Correlation analysis of 25-hydroxyvitamin D against weight, blood pressure, and certain biochemical parameters among study participants.

	Vitamin D 25(OH)D (ng/ml)		
	Pearson correlation (*r*)	*P* value	*N*
VDBP (ng/ml)	−0.421	<0.001	500
(IPTH) (pg/ml)	−0.568	<0.001	500
Corrected ionized calcium (mmol/l)	0.622	<0.001	500
Phosphorous (mmol/l)	0.199	0.102	500
Albumin (g/dl)	0.110	0.114	500
Creatinine (mmol/l)	0.057	0.205	500
IL 10 (pg/ml)	0.575	<0.001	500
TNF-alpha (pg/ml)	−0.300	<0.001	500
IFN-gamma (pg/ml)	−0.356	<0.001	500
Body weight (kg)	−0.012	0.786	500
DBP (mm/Hg)	0.037	0.414	500
SBP (mm/Hg)	−0.092	0.039	500
Age	0.043	0.337	500

*P* value <0.05 = statistically significant, SBP = systolic blood pressure, DBP = diastolic blood pressure, IPTH = intact parathyroid hormone, VDBP = vitamin D binding protein, IL-10 = interleukin-10, TNF-alpha = tumor necrosis factor-alpha, and IFN-gamma = interferon-gamma.

**Table 3:** Binary logistic regression analysis predicted the odds ratio for risk factors of vitamin D deficiency among study participants.

Variables	Frequency (*n* = 500)	Univariate (95% CI)	*P* value	Multivariate (95% CI)	*P* value
Knowledge of vit D foods					
Yes	32	Referent		Referent	
No	468	2.93 (0.15–0.80)	0.014	4.39 (1.69–11.44)	0.002

Milk intake					
Not taken	68	Referent		Referent	
Daily	31	0.49 (0.28–0.84)	0.009	1.77 (0.92–3.14)	0.088
Weekly	152	0.94 (0.41–2.22)	0.824	2.63 (1.05–6.59)	0.040
Monthly	249	0.95 (0.41–2.22)	0.902	2.11 (1.29–3.47)	0.003

Salmon (oily fish)					
Not taken		—		—	
Daily	185	Referent		Referent	
Weekly	221	2.08 (1.38–3.12)	<0.001	1.78 (1.09–2.90)	0.022
Monthly	94	2.43 (1.46–4.04)	0.001	1.96 (1.06–3.64)	0.032

Fruit/vegetable intake					
Not taken		—		—	
Daily	50	Referent		Referent	
Weekly	254	2.12 (1.01–4.44)	0.046	2.45 (1.10–5.43)	0.028
Monthly	196	6.32 (2.98–13.37)	<0.001	7.02 (3.08–16.05)	<0.001

Interleukin-10					
Normal (<10 pg/mL)	419	Referent		Referent	
High (≥10 pg/mL)	81	3.47 (1.97–6.13)	<0.001	3.15 (1.65–6.01)	<0.001

Interferon-gamma					
Normal (<8 pg/mL)	155	Referent		Referent	
High (≥8 pg/mL)	345	13.10 (8.53–20.12)	0.001	10.77 (6.08–19.06)	<0.001

*P* value <0.05 = statistically significant; vit D = vitamin D; for multivariate analysis all other factors such as hemodynamics, biochemistry parameters, age, and weight were adjusted.

## Data Availability

The datasets supporting the conclusions of this article are included within the article and its additional file.

## Supplementary Materials

Supplementary Materials Figure S1: flowchart of the protocol for the selection of subject. Table S1: geographical study sites and their respective coordinates. Table S2: sampling technique method. Table S3: association between predisposing factors for vitamin D deficiency and the stratified geographical areas. Table S4: binary logistic regression analysis predicting the odds ratio for sociodemographics with respect to vitamin D deficiency among study participants.
