# Supplementary material for: Vitamin D Deficiency Is Common in Ghana despite Abundance of Sunlight: A Multicentre Comparative Cross-Sectional Study
Source: J Nutr Metab. 2021 Jun 10;2021:9987141. doi: 10.1155/2021/9987141 (PMC8213472; doi:10.1155/2021/9987141)
Supplement: Supplementary Materials — Figure S1: flowchart of the protocol for the selection of subject. Table S1: geographical study sites and their respective coordinates. Table S2: sampling technique method. Table S3: association between predisposing factors for vitamin D deficiency and the stratified geographical areas. Table S4: binary logistic regression analysis predicting the odds ratio for sociodemographics with respect to vitamin D deficiency among study participants. [file 9987141.f1.docx]

**
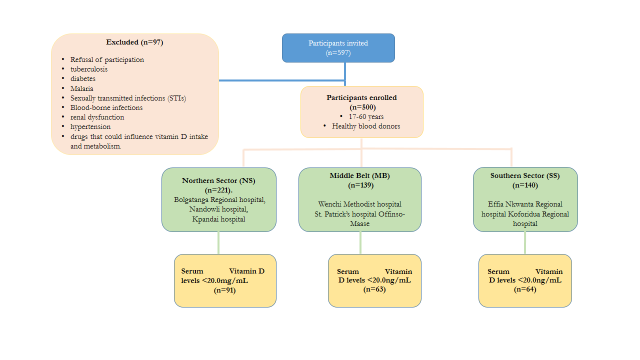
**

**Figure S1. Flowchart of the protocol for the selection of subject**

**Table S1: Geographical study sites and their respective coordinates**

| **Hospital** | **Region** | **Geographical Coordinates** | **Geographical area** |
| --- | --- | --- | --- |
| Bolgatanga regional hospital | Upper East | 10.70^o^N,0.98^o^W | Northern Sector(NS) |
| Nandowli regional hospital | Upper West | 10.25^o^N,2.15^o^W | Northern Sector(NS) |
| Kpandai hospital | Northern | 9.54^o^N,0.91^o^W | Northern Sector(NS) |
| Wenchi-Methodist hospital | Brong-Ahafo | 6.79^o^N,1.68^o^W | Middle Belt (MB) |
| St. Patrick Hospital-Offinso Maase | Ashanti | 6.75oN,1.52^o^W | Middle Belt (MB) |
| Koforidua regional hospital | Eastern | 6.24^o^N,0.45^o^W | Southern Sector (SS) |
| Effia-Nkwata regional hospital | western | 5.39^o^N,2.15oW | Southern Sector (SS) |

| **Site** | | **N** | **n** | **J^th^ term** |
| --- | --- | --- | --- | --- |
| **NS** | Bolgatanga regional hospital | 300 | 71 | 4 |
|  | Nandowli hospital | 300 | 71 | 4 |
|  | Kpandai hospiatal | 500 | 80 | 6 |
| **MB** | Wenchi Methodist hospital | 300 | 70 | 4 |
|  | St. Patrick hospital | 500 | 69 | 7 |
| **SS** | Effia Nkwanta regional hospital | 300 | 69 | 4 |
|  | Koforidua reginal hospital | 300 | 70 | 4 |
| **Total** |  |  | **500** |  |

**Table S2: Sampling Technique Method**

***NS=Northern sector, MB=Middle belt, SS=Southern sector, N= number of participants who qualified for inclusion, n= number of selected participants, J^th^ term= the selection interval.***

**Table S3: Association between predisposing factors for Vitamin D deficiency and the stratified geographical areas**.

| **Variables** | **NS**  **(n=221)** | **MB**  **(n=139)** | **SS**  **(n=140)** | **P-value** |
| --- | --- | --- | --- | --- |
| **Knowledge on Vit D** **foods** |  |  |  |  |
| Yes | 17(53.1%) | 3(9.4%) | 12(37.5%) | 0.926 |
| No | 204(43.6%) | 136(29.1%) | 128(27.4%) | 0.926 |
| **Milk intake** |  |  |  |  |
| Not taken | 27(39.7%) | 37(54.4%) | 4(5.9%) | <0.001 |
| Daily | 15(48.4%) | 13(41.9%) | 3(9.7%) | 0.039 |
| Weekly | 63(41.4%) | 17(11.2%) | 72(47.4%) | <0.001 |
| Monthly | 118(46.6%) | 72(28.9%) | 61(24.5%) | 0.173 |
| **Salmon(Oily fish)** |  |  |  |  |
| Not Taken | - | - | - |  |
| Daily | 83(44.9%) | 53(28.6%) | 49(26.5%) | 0.841 |
| Weekly | 97(43.9%) | 63(28.5%) | 61(27.6%) | 0.287 |
| Monthly | 41(43.6%) | 23(24.5%) | 30(31.9%) | 0.576 |
| **Fruit/vegetable intake** |  |  |  |  |
| Not Taken | - | - | - |  |
| Daily | 24(48.0%) | 16(32.0%) | 10(20.0%) | 0.406 |
| Weekly | 117(46.1%) | 68(26.8%) | 69(27.2%) | 0.694 |
| Monthly | 80(40.8%) | 55(28.1%) | 61(31.1%) | 0.761 |
| **Interleukin 10** |  |  |  |  |
| Normal (<10pg/mL) | 186(44.4%) | 117(27.9%) | 116(27.7%) | 0.938 |
| High (≥10pg/mL) | 35(43.2%) | 22(27.2%) | 24(29.6%) | 0.938 |
| **Interferon Gamma** |  |  |  |  |
| Normal (<8pg/mL) | 79(51.0%) | 44(28.4%) | 32(20.6%) | 0.350 |
| High (≥8pg/mL) | 142(41.2%) | 95(27.5%) | 108(31.3%) | 0.350 |

***P-value<0.05=statistically significant, NS=Northern Sector, MB=middle Belt, SS=Southern Sector, Vit D= Vitamin D.***

**Table S4: Binary logistic regression analysis predicting the odds ratio for socio-demographics with respect to vitamin D deficiency among study participants**

| **Socio-demographics** | **Total(n=500)** | **Univariate**  **(95%CI)** | **P-value** |
| --- | --- | --- | --- |
| **Age group(years)** |  |  |  |
| <20 | 84(16.8%) | 1 |  |
| 20-29 | 239(47.8%) | 1.3(0.8-2.2) | 0.250 |
| 30-39 | 121(24.2%) | 0.8(0.4-1.4) | 0.443 |
| ≥40 | 56(11.2%) | 0.9(0.5-1.8) | 0.779 |
| **Gender** |  |  |  |
| Male | 362(72.4%) | 1 |  |
| Female | 138(27.6%) | 0.9(0.6-1.3) | 0.523 |
| **Marital Status** |  |  |  |
| Single | 309(61.8%) |  |  |
| Married | 180(36.0%) | 0.8(0.6-1.2) | 0.798 |
| Divorced | 11(2.2%) | 0.4(0.1-1.7) | 0.438 |
| **Religious Status** |  |  |  |
| Christians | 376(75.2%) | 1 |  |
| Muslims | 97(19.4%) | 1.1(0.7-1.6) | 0.828 |
| Traditionalists | 27(5.4%) | 1.2(0.6-2.7) | 0.609 |
| **Educational background** |  |  |  |
| None | 69(13.8%) | 1 |  |
| Basic | 76(15.2%) | 0.9(0.4-1.7) | 0.644 |
| Secondary | 207(41.4%) | 1.4(0.8-2.4) | 0.266 |
| Tertiary | 148(29.6) | 1.1(0.6-1.9) | 0.855 |

***P-value<0.05=statistically significant***
